# Triptolide Inhibited Cytotoxicity of Differentiated PC12 Cells Induced by Amyloid-Beta_25–35_ via the Autophagy Pathway

**DOI:** 10.1371/journal.pone.0142719

**Published:** 2015-11-10

**Authors:** Pengjuan Xu, Zhigui Li, Hui Wang, Xiaochen Zhang, Zhuo Yang

**Affiliations:** 1 School of Integrative Medicine, Tianjin University of Traditional Chinese Medicine, Tianjin, 300193, China; 2 College of Medicine, Tianjin Key Laboratory of Tumor Microenvironment and Neurovascular Regulation, State Key Laboratory of Medicinal Chemical Biology, Nankai University, Tianjin, 300071, China; 3 College of Life Science, Nankai University, Tianjin, 300071, China; University of Pecs Medical School, HUNGARY

## Abstract

Evidence shows that an abnormal deposition of amyloid beta-peptide_25–35_ (Aβ_25–35_) was the primary cause of the pathogenesis of Alzheimer’s disease (AD). And the elimination of Aβ_25–35_ is considered an important target for the treatment of AD. Triptolide (TP), isolated from *Tripterygium wilfordii Hook*.*f*. (TWHF), has been shown to possess a broad spectrum of biological profiles, including neurotrophic and neuroprotective effects. In our study investigating the effect and potential mechanism of triptolide on cytotoxicity of differentiated rat pheochromocytoma cell line (the PC12 cell line is often used as a neuronal developmental model) induced by Amyloid-Beta_25–35_ (Aβ_25–35_), we used 3-(4, 5-dimethylthiazol-2-yl)-2, 5- diphenyltetrazolium bromide (MTT) assay, flow cytometry, Western blot, and acridine orange staining to detect whether triptolide could inhibit Aβ_25–35_–induced cell apoptosis. We focused on the potential role of the autophagy pathway in Aβ_25–35_-treated differentiated PC12 cells. Our experiments show that cell viability is significantly decreased, and the apoptosis increased in Aβ_25–35_-treated differentiated PC12 cells. Meanwhile, Aβ_25–35_ treatment increased the expression of microtubule-associated protein light chain 3 II (LC3 II), which indicates an activation of autophagy. However, triptolide could protect differentiated PC12 cells against Aβ_25–35_-induced cytotoxicity and attenuate Aβ_25–35_-induced differentiated PC12 cell apoptosis. Triptolide could also suppress the level of autophagy. In order to assess the effect of autophagy on the protective effects of triptolide in differentiated PC12 cells treated with Aβ_25–35_, we used 3-Methyladenine (3-MA, an autophagy inhibitor) and rapamycin (an autophagy activator). MTT assay showed that 3-MA elevated cell viability compared with the Aβ_25–35_-treated group and rapamycin inhibits the protection of triptolide. These results suggest that triptolide will repair the neurological damage in AD caused by deposition of Aβ_25–35_ via the autophagy pathway, all of which may provide an exciting view of the potential application of triptolide or TWHF as a future research for AD.

## Introduction

Alzheimer’s disease (AD) is a neurodegenerative disease and it is the main cause of dementia. AD accounts for 60%-70% of dementia syndromes in people older than 65 years [1]. Clinically, the major characteristic of AD is memory impairment, especially impairment of episodic memory [2]. Evidence shows that an abnormal deposition of amyloid beta-peptide_25–35_ (Aβ_25–35_) could cause the loss of neurons, and synaptic lesions, all of which are considered to affect the memory. Therefore, the abnormal deposition of Aβ_25–35_ in plaques of brain tissue was the primary cause of the pathogenesis of AD [3]. Thus, the prevention and elimination of excessive accumulation of Aβ_25–35_ is considered an important target for the treatment of AD [4–6].

Autophagy is widely present in eukaryotic cells. It is an evolutionarily conserved catabolic process involved in the elimination and recycling of non-essential or abnormal organelles and long-lived proteins by lysosomes [7]. Since De Duve first used the word autophagy 50 years ago, people mainly use it to describe the digestion/degradation of misfolded or aggregated proteins and damaged organelles in the cells [8, 9]. With the development of technology and medicine, many researchers found that disrupting the autophagy is the target for many diseases such as pancreatic cancer and non-small cell lung cancer. In addition, numerous studies using postmortem human tissue, genetic- and toxin- induced animal and cellular models confirmed that many etiological factors of neurodegenerative disorders can perturb the autophagic process. More recent data support the view that autophagy is critical for the pathogenesis of neurodegenerative disorders [10–12]. More and more evidence shows the pathophysiological roles of autophagy in neurodegenerative disorders, such as Alzheimer’s disease, Parkinson’s disease, Huntington’s disease and Amyotrophic lateral sclerosis [13]. For example, in Parkinson’s disease, the autophagic pathway is involved in mitochondrial dysfunction, which serves as a crucial pathogenic mechanisms. Mitochondrial dysfunction is the primary mediator of cell death in Parkinson’s disease [14]. In Huntington’s disease, post mortem brains of patients with Huntington's disease have endosomal and/or lysosomal organelles and multivesicular bodies, characteristic features of autophagy [15]. Excessive autophagy have also been examined in lymphoblasts of Huntington's disease patients’ tissue [16]. Furthermore, an increase in autophagic activity can affect the formation and degradation of β-amyloid and tau protein [17]. Autophagy is now recognized as an arbiter of neuron survival and apoptosis [18] in neurodegenerative disorders [19]. An increasing amount of data confirms that autophagy could increase the accumulation of intracellular Aβ_25–35_ protein aggregates in neurons [20, 21]. In addition, there are many autophagosomes in the brain in early AD [22]. Therefore, autophagy has been the direction of research in Aβ_25–35_ deposition in recent years. Meanwhile, reactive oxygen species (ROS) are closely associated with oxidative stress, including superoxide anion (O^2 -^), hydroxyl radical (• OH), and hydrogen peroxide (H_2_O_2_) [23]. The latest research suggests that ROS as signaling molecules could increase production of mitochondrial membrane lipid peroxidation and mitochondrial dysfunction, causing autophagic cell death [24, 25]. Accordingly, clarifying the molecular signal mechanism of autophagy in Aβ-induced cytotoxicity will contribute to discovering the treatment target of AD.

*Tripterygium wilfordii Hook*.*f*. (TWHF) is a representative Traditional Chinese Medicine (TCM) herb, which belongs to the Celastraceae family [26]. TWHF has aided in treating inflammatory and autoimmune diseases for hundreds of years [27, 28]. According to the data, more than 300 compounds were identified in TWHF, such as triptolide, tripdiolide, tripcheorolide, 16-hydroxytriptolide, triptonide, and triptriolide [29]. As one kind of triepoxide lactone, triptolide (TP) is the major active compound in TWHF [30], which has been shown to have a broad spectrum of biological profiles including anti-inflammatory, immunosuppressive, anti-fertility, anti-tumor activity, neurotrophic and neuroprotective effects [26]. Currently, much of the available data reveals that triptolide is a promising immunosuppressive and anti-inflammatory agent [31]. In addition, triptolide could easily penetrate the blood-brain barrier because of its lipophilic character and small molecular size [32, 33], and it is proven to have a potential medicinal effect for diseases of the central nervous system (CNS) such as Parkinson's disease, spinal cord and brain injuries, and multiple sclerosis [26]. Gu and He *et al* point out that triptolide plays a neuroprotective role in a variety of cell models. For example, triptolide could decrease the Ca^2+^ concentration that is induced by Aβ_25–35_ [34]. Moreover, triptolide inhibits the apoptosis of PC12 cells treated with glutamate [35]. However, little is known about whether triptolide has a protective effect on cytotoxicity of differentiated PC12 cells induced by Aβ_25–35_ and what the mechanisms are.

Based on these, the purpose of this study was to assess whether triptolide could protect against Aβ induced cytotoxicity in differentiated PC12 cells. In our experiments, we use MTT assay and flow cytometry to investigate the protective effects of triptolide. Western blot and acridine orange staining were chosen to detect the mechanism of triptolide on differentiated PC12 cells treated with Aβ_25–35_. All of these may provide an interesting view of the potential application of triptolide or TWHF in future research for AD.

## Materials and Methods

### Materials

Aβ_25–35_, 3-(4, 5-dimethylthiazol-2-yl)-2, 5-diphenyltetrazolium bromide (MTT), triptolide, rapamycin and 3-Methyladenine (3-MA) were purchased from Sigma Chemical Co., MO, USA. The RPMI 1640 medium and fetal bovine serum (FBS) were purchased from Gibco BRL, USA. The Annexin V-FITC propidium iodide (PI) apoptosis detection kit was from Bipec Biopharma Corporation, USA. The ROS testing kit was from Genmed Scientifics Inc., USA. Mouse monolyclonal anti-LC 3 antibodies (primary antibody, working dilution 1:1000) were purchased from Medical & Biological Laboratories Co., Ltd. and mouse polyclonal anti-β-actin IgG (primary antibody, working dilution 1:1000) were obtained from Santa Cruz Biotechnology, Inc. CA, U.S.A. The Alexa 594-conjugated goat anti-mouse IgG secondary antibody was obtained from Invitrogen, San Diego, CA, USA. Chemiluminescent HRP substrate (Immobilon western) was purchased from Millipore Corporation, Billerica, MA, U.S.A.

### Pretreatment of Aβ_25–35_ and triptolide

Aβ_25–35_ (molecular formula: C_45_H_81_N_13_O_14_S, molecular weight: 1060.27, purity: ≥97%) was purchased from Sigma. Aβ_25–35_ was diluted to 1mmol/L with phosphate buffered saline (PBS), and incubated at 37°C for 2 weeks to induce the aggregation of Aβ_25–35._ When using, it was diluted to different concentrations with RPMI 1640 medium.

Triptolide (PG490, molecular formula: C_20_H_24_O_6_, molecular weight: 360.4) was purchased from Sigma. The material was composed of white to off-white crystals, had a melting point of 235–237°C, and conformed to standard triptolide preparation by proton nuclear magnetic resonance. The material was 98% pure by reverse phase high pressure liquid chromatography evaluation. Before using, triptolide was soluble in dimethylsulfoxide (DMSO). After reconsititution, triptolide was stored at -20°C at a concentration of 1 mg/mL. When using, it was diluted to different concentrations with RPMI 1640 medium.

### Cell culture

The rat pheochromocytoma cell line (PC12, derived from the American Type Culture Collection) was purchased from the Institute of Basic Medical Sciences Chinese Academy of Medical Sciences. It has been described in our previously work [23, 36]. The cell line was derived from a rat adrenal medulla pheochromocytoma. In the presence of nerve growth factor (NGF), the undifferentiated PC12 cells could differentiate into sympathetic-like neurons, which were widely used as the model of neurons *in vitro* [37].

The undifferentiated PC12 cells were cultured in an incubator aerated with 95% humidified air with 5% CO_2_ at 37°C, supplemented with 10% FBS, 5% horse serum, and 1% antibiotics (penicillin and streptomycin). Then the medium was replaced with serum-free RPMI1640 supplemented with 50 ng/mL NGF for 7 days to obtain neuronal differentiated PC12 cells. Then differentiated PC12 cells were cultured in RPMI 1640 medium (pH = 7.4) supplemented with 5% FBS and 1% antibiotics (penicillin and streptomycin). Cells were grown at 37°C in 95% humidified air with 5% CO_2_. All subsequent experiments in the present study were undertaken with these differentiated PC12 cells.

### Cytotoxicity induced by Aβ_25–35_ on differentiated PC12 cells

*In vitro* cytotoxicity induced by Aβ_25–35_ on differentiated PC12 cells was assessed by the MTT assay, which was widely used to evaluate the cytotoxic activity. Differentiated PC12 cells were cultured on 96-well plates with RPMI 1640 medium for stabilization. 24 hours later, cells were incubated with different concentrations of Aβ_25–35_ (5, 10, 20 μmol/L) for 24 hours. Subsequently, MTT was added and incubated for 4 hours at 37°C. After that, formazan crystals were dissolved by DMSO and measured at a wavelength of 570 nm. The cell viability was expressed as a percentage of viability of the control culture. Each condition and experiment was repeated three times.

### The viability of differentiated PC12 cells treated with different concentrations of triptolide

After differentiated PC12 cells were cultured on 96-well plates with RPMI 1640 medium for stabilization, differentiated PC12 cells were incubated with different concentrations of triptolide (10^−11^, 10^−10^, 10^−9^ mol/L) for 24 hours. The concentrations in this study were chosen according to the published data [26, 35]. Then cell viability was determined by the MTT assay. Each condition and experiment was repeated three times.

### Effect of different concentrations of triptolide on Aβ_25–35_-induced cytotoxicity in differentiated PC12 cells

In the study, we chose 10 μmol/L Aβ_25–35_ as a final concentration to incubate differentiated PC12 cells to detect the cytotoxicity of Aβ. In order to assess the effect of triptolide on Aβ_25–35_-induced cytotoxicity in PC12 cells, our experiment was performed with the following treatments: control (culture medium), 10 μmol/L Aβ_25–35_, 10 μmol/L Aβ_25–35_+10^−11^ mol/L triptolide, 10 μmol/L Aβ_25–35_+10^−10^ mol/L triptolide, and 10 μmol/L Aβ_25–35_+10^−9^ mol/L triptolide. Each group of cells was cultured for 24 hours. Then cell viability was determined by the MTT assay. Each condition and experiment was repeated three times.

### Detection of apoptotic cells

Annexin V-FITC and PI staining analyzed by flow cytometry (Beckman-Coulter, USA) was used to detect the apoptotic index. The cells were plated in 6-well plates and exposed to 10 μmol/L Aβ_25–35_ and /or 10^−10^ mol/L triptolide or cell culture medium without treatment (control) for 24 hours. Then cells were harvested and rinsed with PBS. After that, cells were re-suspended in 400 μL 1×binding buffer containing 10 μL PI and 5 μL V-FITC, and incubated for 15 min at room temperature in the dark. The cell suspension was determined by flow cytometry to analyze the apoptotic rate. The apoptosis ratio was calculated as follows: apoptosis ratio (%) = (the percentage of early apoptotic cells) + (the percentage of late apoptotic cells). The percentage of the cells is presented in the area of respective quadrant profiles. All experiments were performed a minimum of three times.

### Measurement of ROS generation

The level of ROS induced by different conditions was measured by dichlorodihydrofluorescein diacetate (DCFH-DA). Cells were exposed to cell culture medium, 10 μmol/L Aβ_25–35,_ and 10 μmol/L Aβ_25–35_+10^−10^ mol/L triptolide for 24 hours. Then differentiated PC12 cells were incubated in the staining solution containing DCFH-DA for 20 min at 37°C and washed with PBS. The methodology followed the procedures as described in the ROS assay kit. The intracellular accumulation of ROS was measured by flow cytometry. The level of ROS generation was calculated as follows: the level of ROS (%) = the percentage of DCF-positive cells in M1 region. All experiments were performed a minimum of three times.

### Acridine orange staining

Cellular acidic compartments were examined by acridine orange staining. Acridine orange (molecular formula: C_17_H_19_N_3_·HCl·ZnCl_2_, molecular weight: 438.1, purity:≥98%) was diluted to 1μmol/L with PBS. Differentiated PC12 cells were seeded in 6-well plates and treated with cell culture medium, 10 μmol/L Aβ_25–35_ and /or 10^−10^ mol/L triptolide for 24 hours. After treatment, the cells were stained with acridine orange at 37°C for 15 min. After washing three times with PBS, the cells were immediately visualized by a Leica TCS SP5 laser-scanning confocal microscope for the detection of acidic vesicular organelles. For the quantitation analysis, fluorescent intensity was quantified using Image-Pro Plus 6.0 (IPP 6.0) [38]. Data were analyzed from several cells of one sample, and there were seven samples for each group. The TIFF images were not processed before measurement of signal intensities.

### Immunofluorescence

The cells were treated with cell culture medium, 10 μmol/L Aβ_25–35_, 10 μmol/L Aβ_25–35_+10^−10^ mol/L triptolide and 10^−10^ mol/L triptolide for 24 hours. Following a 30 min-fixation in 4% paraformaldehyde at 4°C, cells were washed with PBS, and then were permeabilized with 0.5% Triton X-100 and blocked with 10% NGS for 2 hours at room temperature. After that, cells were incubated with mouse anti-LC3 antibody as the primary antibody overnight at 4°C. After washing with PBS, cells were incubated with Alexa 594-conjugated anti-mouse IgG for 3 hours at room temperature. Thereafter, the cell nuclei were stained by DAPI. The fluorescent signals were examined using an Olympus FV1000 laser-scanning confocal microscope, and fluorescent intensity was quantified using IPP 6.0. Data were analyzed from several cells of one sample, and there were seven samples for each group. The TIFF images were not processed before measurement of signal intensities. All experiments were performed a minimum of three times.

### Western blot assay

Differentiated PC12 cells were incubated with cell culture medium, 10^−10^ mol/L triptolide, 10 μmol/L Aβ_25–35_ and /or 10^−10^ mol/L triptolide for 24 hours. Then cells were lysed for 30 min on ice. Protein samples were centrifuged for 15 min at 4°C. Samples were subjected to electrophoresis on SDS-polyacrylamide gel and the separated proteins were electrotransferred to polyvinylidenedifluoride (nitrocellulose) membranes. Membranes were blocked in 5% non-fat milk for 2 hours at room temperature, and then incubated with primary antibody (anti-LC3) overnight at 4°C. Subsequently, membranes were washed and incubated with a 1:2500 dilution of secondary antibodies for 1 hour at room temperature. The bands were visualized by a chemiluminescent HRP substrate detection kit. For analysis, quantization was performed by scanning and determination of the intensity of the hybridization signals. We used the LC3 II/LC3 I ratio to comprehensive assess autophagy flux. Protein loading and transferring of LC3 II and LC3 I were standardized by preliminary experiments in which β-actin expression on the same Western blot sample was quantitated by use of the Imge J software [39]. All experiments were performed a minimum of three times.

### Effect of 3-MA and rapamycin

In order to assess the effect of autophagy on the protective effects of triptolide in differentiated PC12 cells treated with Aβ_25–35_, we used 3-MA (an autophagy inhibitor, 10 μmol/L) and rapamycin (an autophagy activator, 10ng/mL). PC12 cells were incubated with culture medium, 10 μmol/L Aβ_25–35_, 10 μmol/L Aβ_25–35_+10 μmol/L 3-MA, 10 μmol/L 3-MA, 10ng/mL rapamycin, 10 μmol/L Aβ_25–35_+10^−10^ mol/L triptolide, and 10 μmol/L Aβ_25–35_+10^−10^ mol/L triptolide +10ng/mL rapamycin for 24 hours. The cell viability was determined by MTT. Each condition and experiment was repeated three times.

### Statistical analysis

All of the results were expressed as mean ±S.E.M and analyzed by Origin 8.1 and SPSS 17.0. The results of the groups treated with the triptolide were compared to those of the no- triptolide -treated group and represented as the percentage of the control value. The test of Kolmogorov–Smirnov with the correction of Lilliefors was used to evaluate the normal distribution and the test of Levene to evaluate the homogeneity of variance. Statistical analysis was performed using one-way ANOVA followed by a Turkey’s multiple range test and *P*<0.05 was considered significant.

## Results

### Cytotoxicity induced by Aβ_25–35_ on differentiated PC12 cells

We first tested the cytotoxicity of Aβ_25–35_ on differentiated PC12 cells by MTT assay. Kolmogorov-Smirnov with the correction of Lilliefors and Levene test showed that all of our data satisfied the normal distribution (*P*>0.05) and the homogeneity of variance (*P*>0.05). Therefore, it was omitted in next experiments.

**Fig 1:**
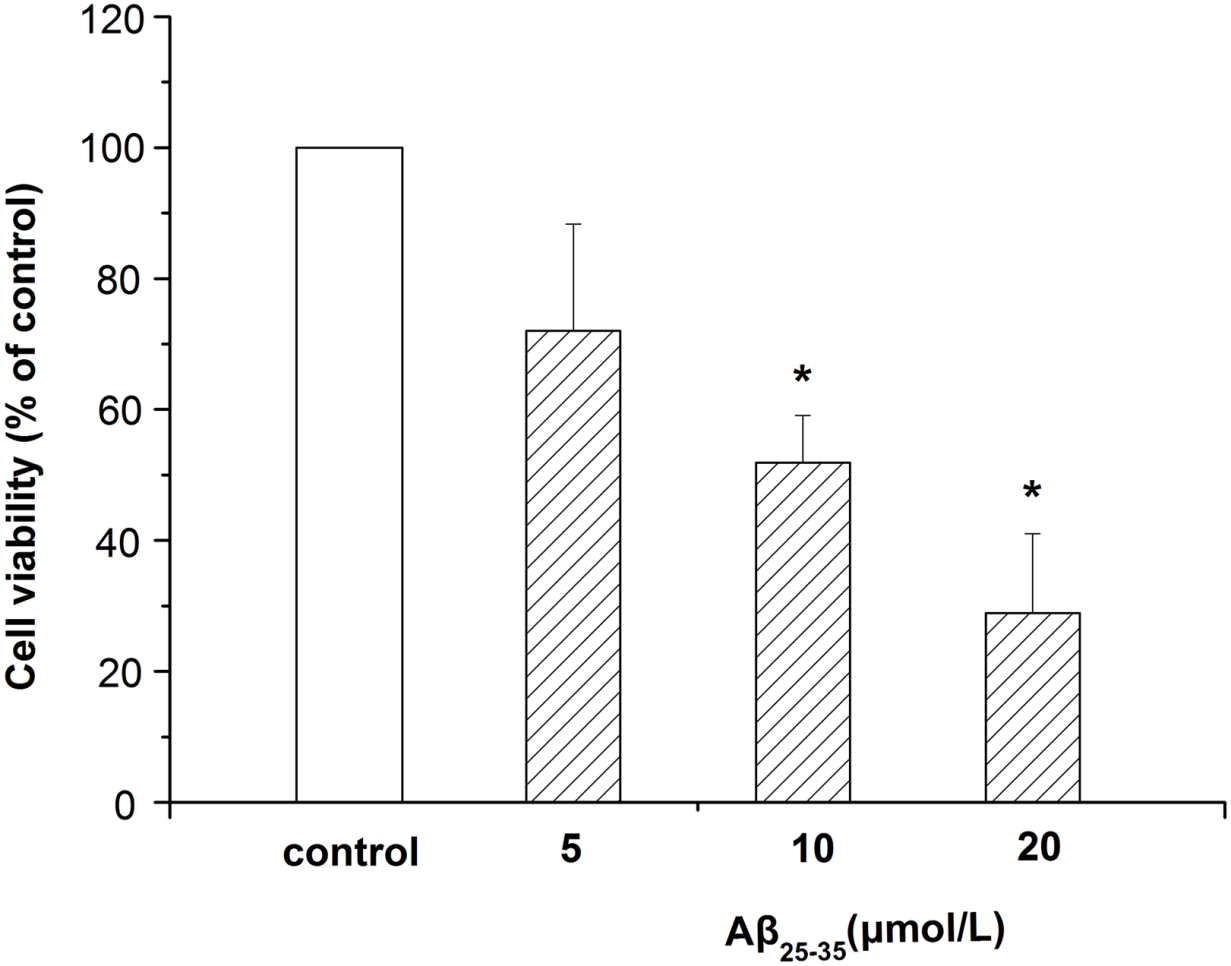
Cytotoxicity induced by Aβ_25–35_ in differentiated PC12 cells. Cells were treated with different concentrations (5, 10, 20 μmol/L) of Aβ_25–35_ for 24 hours. The cell viability was measured by MTT assay. Data represents the mean ± S.E.M. n = 6/group. *P < 0.05 vs. control group.

As shown in Fig 1, exposure of cells to different concentrations of Aβ_25–35_ (5, 10, 20 μmol/L) for 24 hours resulted in a decrease of the cell viability, which indicated that Aβ_25–35_ induced toxicity in differentiated PC12 cells. Additionally, the toxicity induced by Aβ_25–35_ was in a concentration-dependent manner. 10 μmol/L Aβ_25–35_ was chosen in this experiment because of the 50% viability.

### Viability of differentiated PC12 cells treated with different concentrations of triptolide

**Fig 2:**
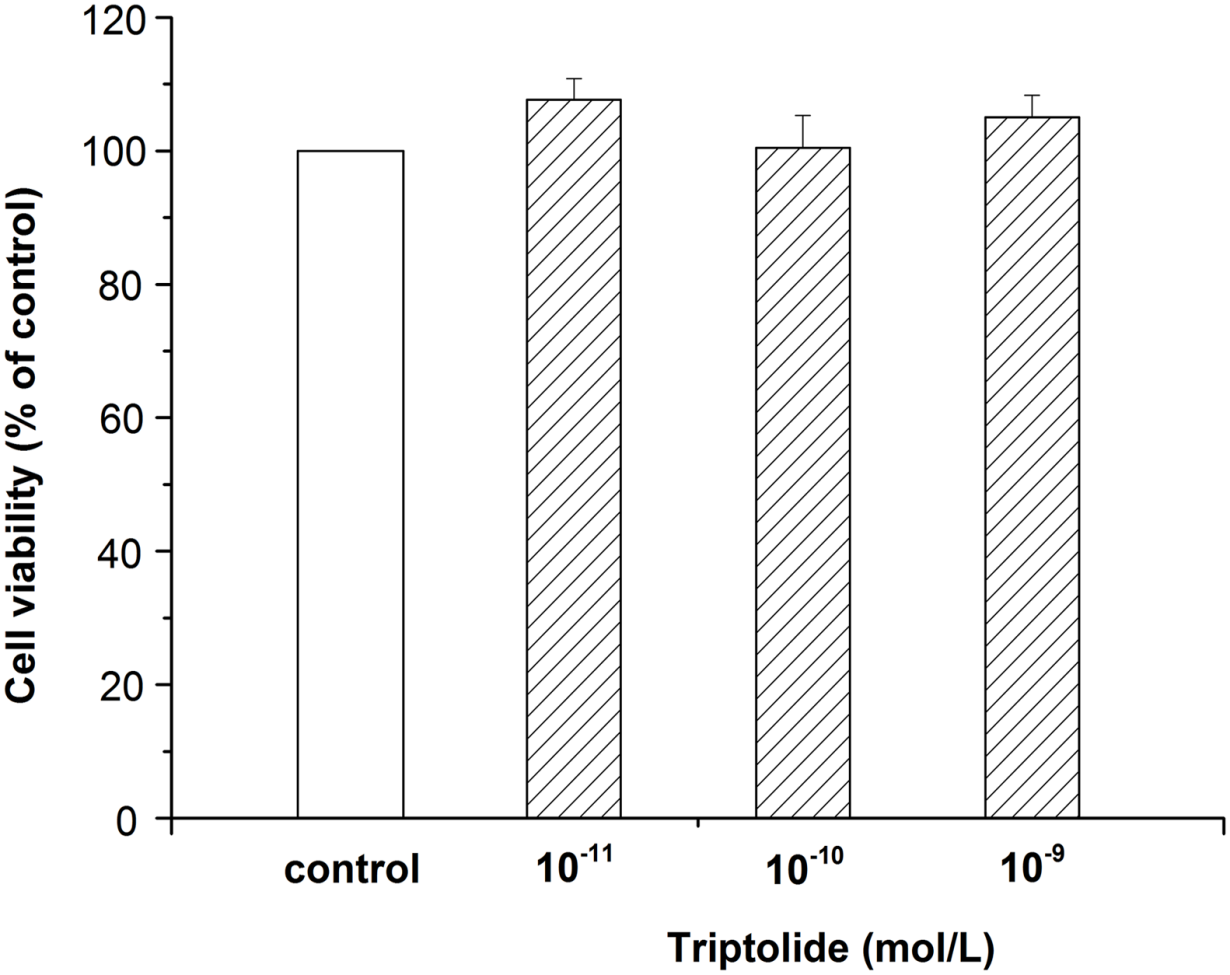
Viability of differentiated PC12 cells treated with different concentrations of triptolide. Differentiated PC12 cells were incubated with different concentrations of triptolide (10^−11^, 10^−10^, 10^−9^ mol/L) for 24 hours. Cell viability was determined by MTT assay. Data represents the mean ± S.E.M. n = 6/group.

Differentiated PC12 cells were treated with different concentrations (10^−11^, 10^−10^, 10^−9^ mol/L) of triptolide for 24 hours, followed by the MTT assay to determine cell viability. As shown in Fig 2, cell viability showed no significant difference with the control group when the concentration changed. The results suggest that our concentrations of the triptolide were safe for differentiated PC12 cells.

### Effect of different concentrations of triptolide on Aβ_25–35_-induced cytotoxicity in differentiated PC12 cells

**Fig 3:**
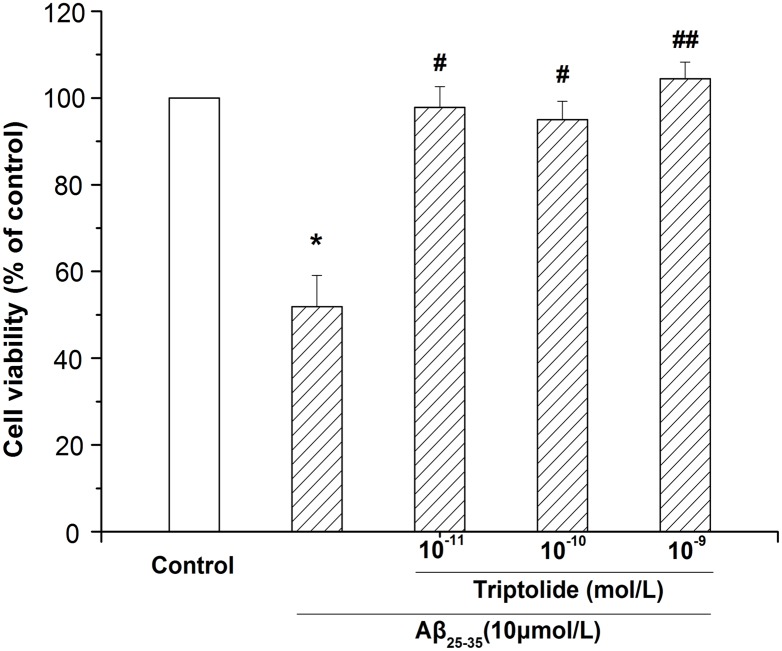
Effect of different concentrations of triptolide on Aβ_25–35_-induced cytotoxicity in differentiated PC12 cells. Differentiated PC12 cells were treated with culture medium, 10 μmol/L Aβ_25–35_, 10 μmol/L Aβ_25–35_+10^−11^ mol/L triptolide, 10 μmol/L Aβ_25–35_+10^−10^ mol/L triptolide, and 10 μmol/L Aβ_25–35_+10^−9^ mol/L triptolide for 24 hours. Cell viability was measured by MTT assay and the results were expressed as the percentile of absorbance of treated samples compared to that of the control. Data represents the mean ± S.E.M. n = 6/group. *P < 0.05 vs. control group. ^#^ *P* < 0.05, ^##^ *P* < 0.01 vs. 10 μmol/L Aβ_25–35_ group.

Differentiated PC12 cells were incubated with different concentrations of triptolide (10^−11^, 10^−10^, 10^−9^ mol/L) in the presence of 10 μmol/L Aβ_25–35_ for 24 hours and MTT assay was used to detect the effect of triptolide. The results in Fig 3 show that Aβ_25–35_ could decrease the cell viability and when treated with triptolide the viability of differentiated PC12 cells was significantly increased. The results indicate that triptolide can alleviate cellular damage caused by Aβ_25–35_, which means that triptolide has a neuroprotective effect.

### Effect of triptolide on Aβ_25–35_-induced apoptosis in differentiated PC12 cells

**Fig 4:**
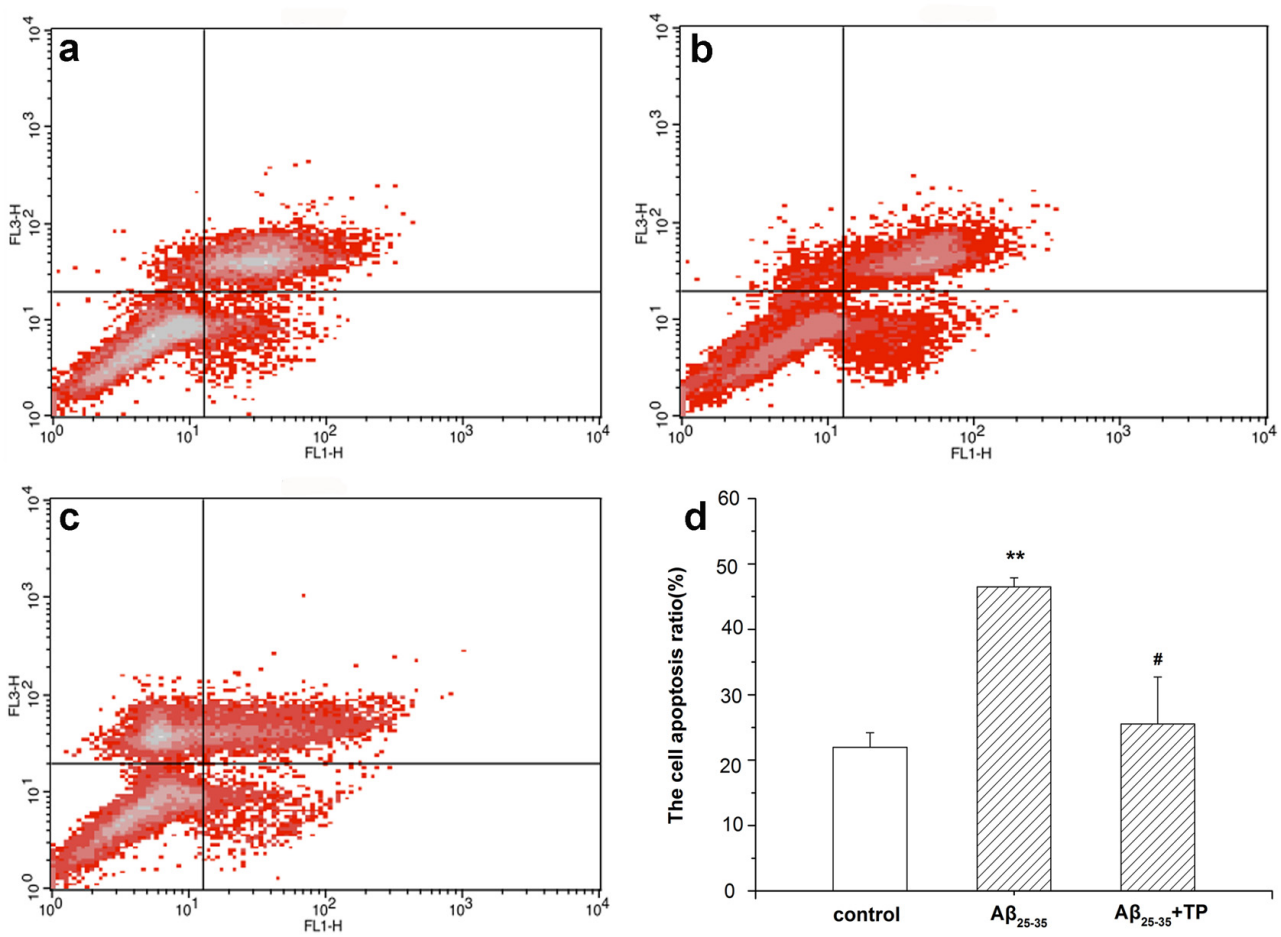
Effect of triptolide on Aβ_25–35_-induced apoptosis in differentiated PC12 cells. The apoptotic rate of differentiated PC12 cells was analyzed by flow cytometry after being incubated with culture medium (a), 10 μmol/L Aβ_25–35_ (b), and 10 μmol/L Aβ_25–35_+10^−10^ mol/L triptolide (c) for 24 hours. The intensity of Annexin V-FITC fluorescence was on the X-axis and PI fluorescence was on the Y-axis. The bar graph was shown in (d). The apoptosis ratio was calculated as follows: apoptosis ratio (%) = (the percentage of early apoptotic cells) + (the percentage of late apoptotic cells). And the percentage of the cells is presented in the area of respective quadrant profiles. Each data represents the mean ± S.E.M. n = 3/group. **P < 0.01 vs. control group. ^#^ *P* < 0.05 vs. 10 μmol/L Aβ_25–35_ group.

The effect of triptolide on Aβ_25–35_-induced apoptosis in differentiated PC12 cells was tested by flow cytometry, as shown in Fig 4. After incubation with 10 μmol/L Aβ_25–35_ for 24 hours, the apoptotic rates of cells were increased significantly compared to those of the control group without treatment (Fig 4d). When treated with 10^−10^ mol/L triptolide and 10 μmol/L Aβ_25–35_ for 24 hours, the apoptotic rate of differentiated PC12 cells was significantly decreased. The results indicate that triptolide has neuroprotective effects on differentiated PC12 cells treated with Aβ_25–35_.

### Measurement of ROS generation

**Fig 5:**
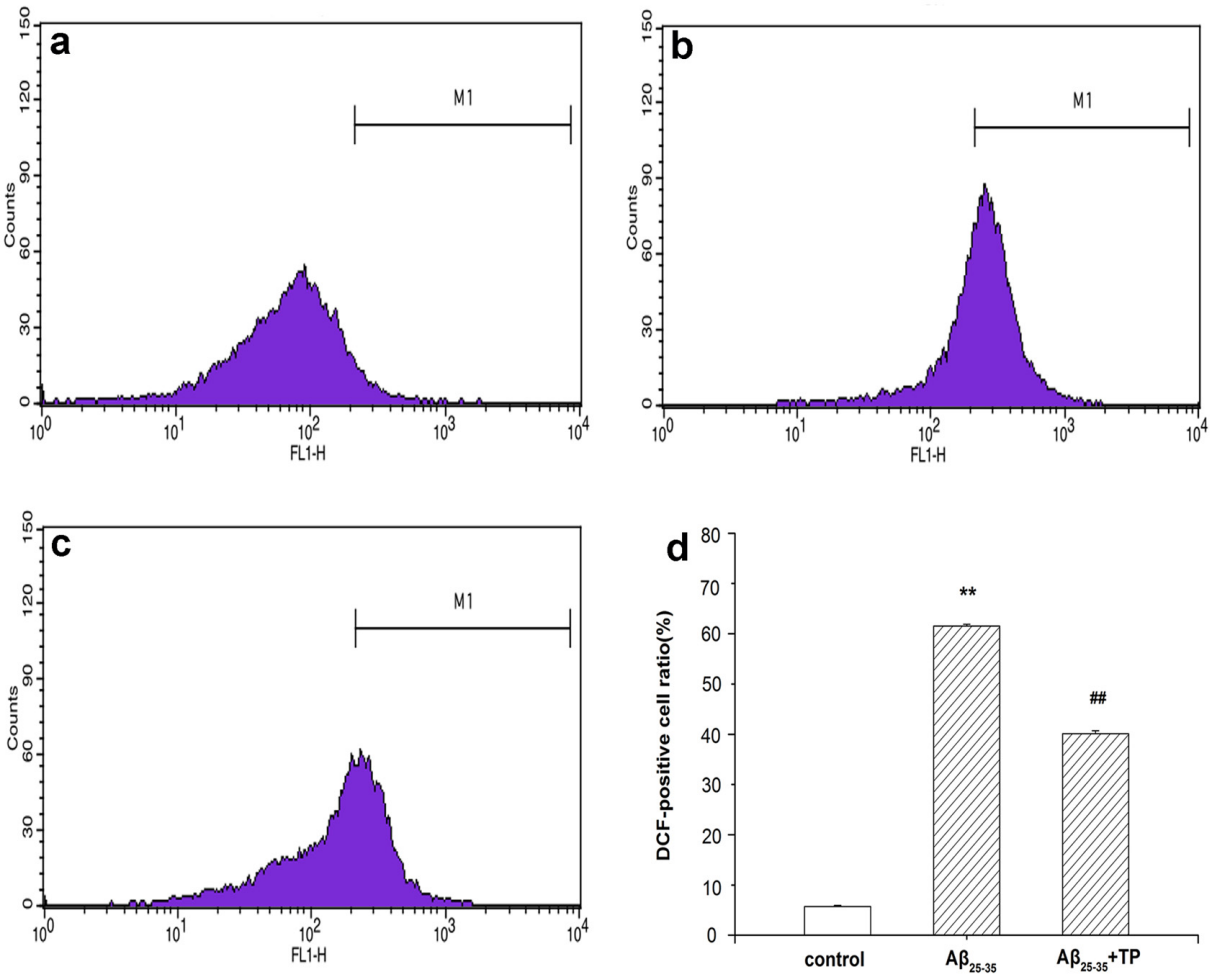
Measurement of ROS generation. ROS levels were determined by flow cytometric analysis. The level of ROS generation was calculated as follows: the level of ROS (%) = the percentage of DCF-positive cell in M1 region. Differentiated PC12 cells were incubated with 10 μmol/L Aβ_25–35_ (b), and 10 μmol/L Aβ_25–35_+10^−10^ mol/L triptolide (c) for 24 hours. Cells were cultured with culture medium (a). The bar graph is shown in (d). Data represents the mean ± S.E.M. n = 3/group. **P < 0.01 vs. control group. ^##^ *P* < 0.01 vs. 10 μmol/L Aβ_25–35_ group.

After treatment with different drugs (cell culture medium, 10 μmol/L Aβ_25–35_ and 10 μmol/L Aβ_25–35_+10^−10^ mol/L triptolide) for 24 hours, ROS was measured by flow cytometry. As shown in Fig 5, Aβ_25–35_ significantly increased ROS levels. When treated with 10^−10^ mol/L triptolide, the ratio of ROS decreased from 61.54% to 40.1%. The results reveal that treatment with triptolide could inhibit the intracellular ROS level induced by Aβ_25–35_ in differentiated PC12 cells.

### Morphological change observations using acridine orange staining

**Fig 6:**
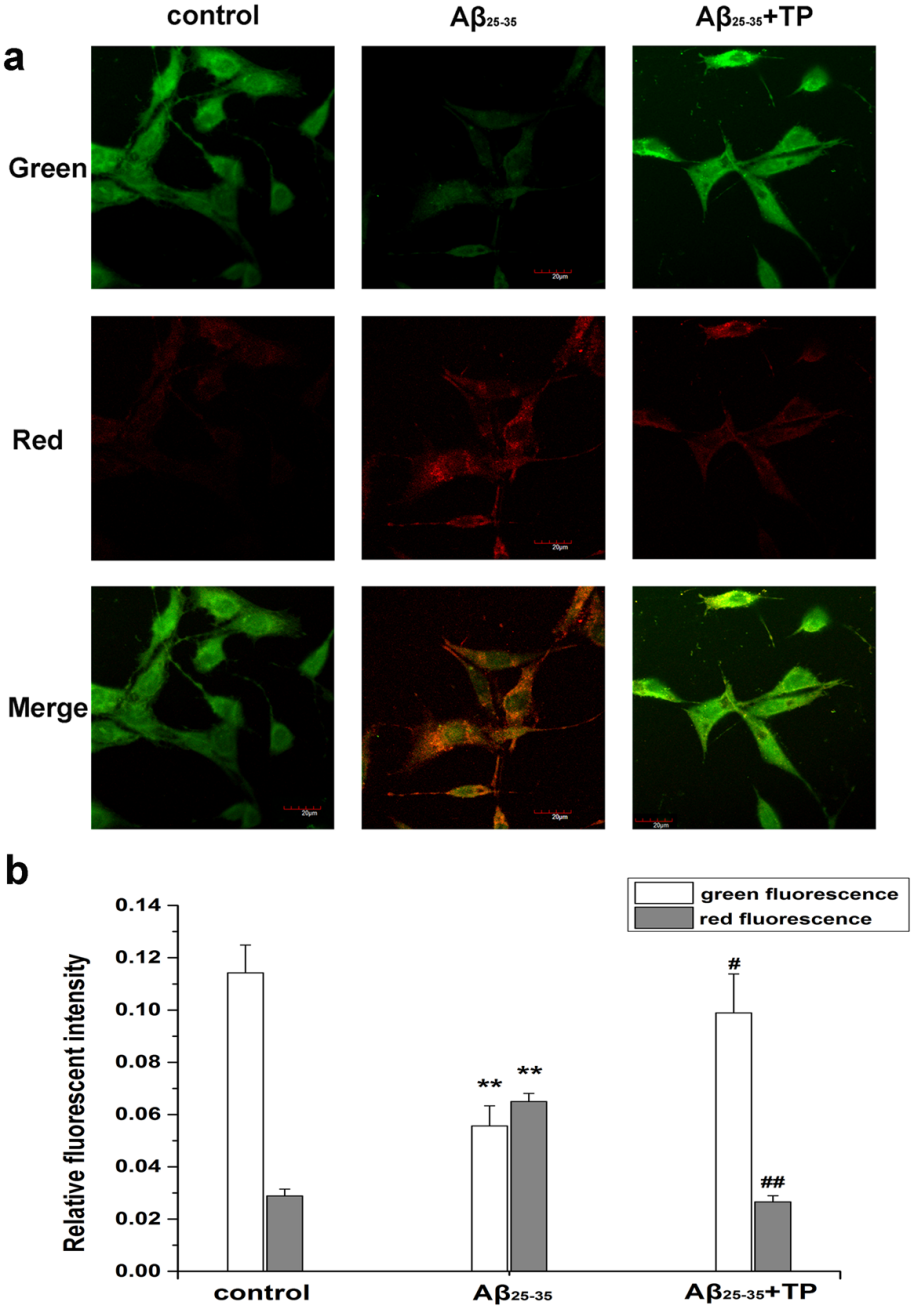
Measurement of the acidic compartment in differentiated PC12 cells. To characterize autophagy, we used acridine orange staining to observe the acidic compartment in differentiated PC12 cells. Cells were incubated with culture medium, 10 μmol/L Aβ_25–35_, 10 μmol/L Aβ_25–35_+10^−10^ mol/L triptolide for 24 hours. Cellular acidic compartments were stained to orange. Fig green cells with the green filter and Fig red with the red filter. Scale bar, 20 μm. Fig b was the corresponding linear diagram of relative fluorescent intensity. Data were presented as the means ±mean ± S.E.M. n = 7/group. **P < 0.01 vs. control group. ^#^ *P* < 0.05, ^##^ *P* < 0.01 vs. 10 μmol/L Aβ_25–35_ group.

Acridine orange is a nucleic acid dye that also enters acidic compartments, such as acidic vesicular organelles, where it becomes protonated and sequestered. Acridine orange staining is often used to detect the occurrence of autophagy. As shown in Fig 6a, green fluorescence with minimal orange fluorescence was displayed in the control group; in the Aβ_25–35_-treated cells the acidic compartments displayed orange fluorescence and green fluorescence with minimal orange fluorescence being displayed in the triptolide-treated cells. Moreover, acidic compartments of Aβ_25–35_-treated differentiated PC12 cells (the level of red fluorescence) were markedly more than triptolide -treated cells (*P*<0.01) in Fig 6b. Our data (the raw data was showed in S1 Table) provide evidence that the number of acidic vesicular organelles increased when the cells were treated with Aβ_25–35_, which means that autophagy processes were activated, and triptolide could inhibit autophagy.

### Morphological evaluation of autophagy

**Fig 7:**
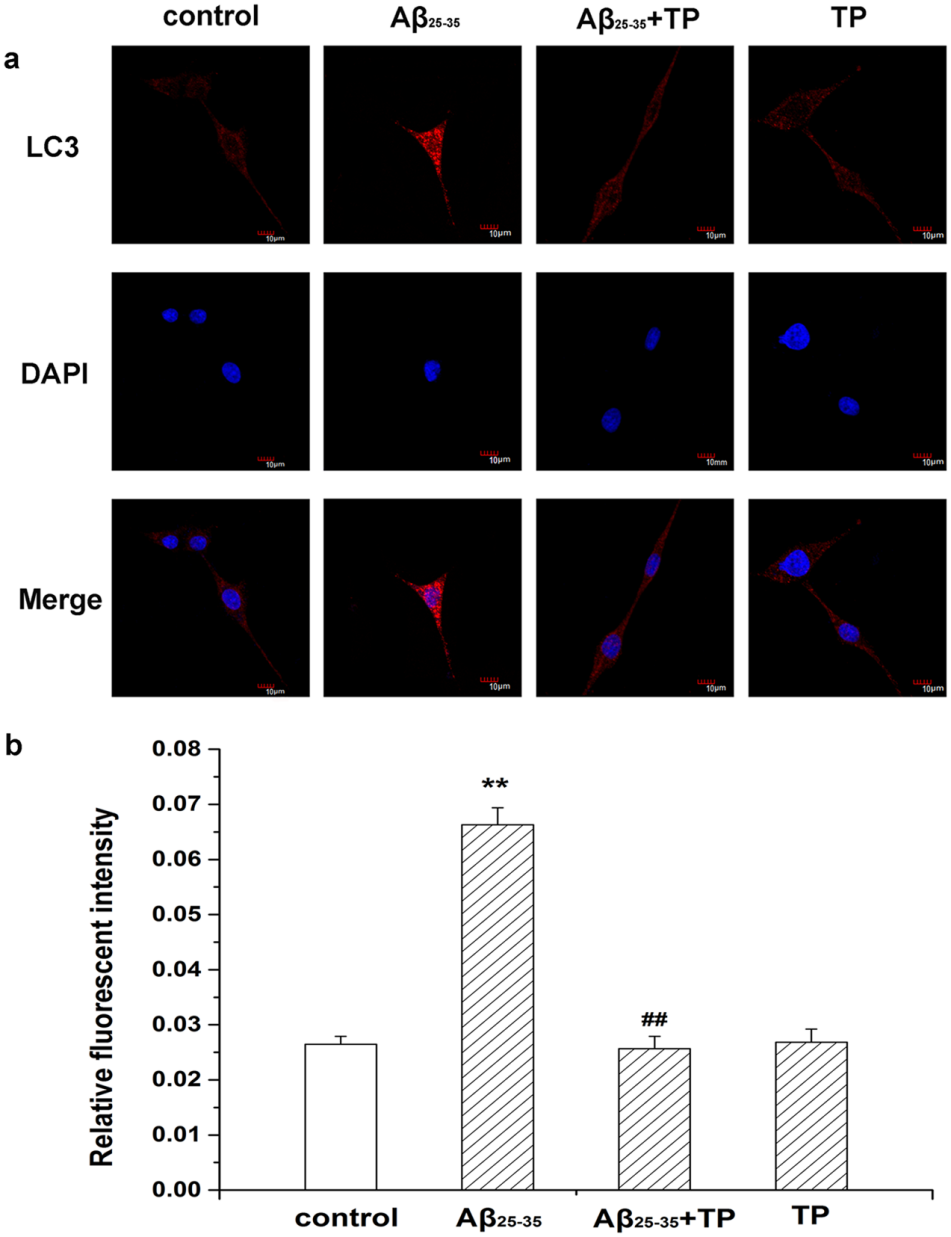
Expression of LC3 in differentiated PC12 cells. Morphological evaluation of autophagy in differentiated PC12 cells by immunofluorescence was acquired by confocal microscopy. The immunohistochemical staining showed that LC3 was expressed in the control group (cells treated with culture medium), 10 μmol/L Aβ_25–35_ group, 10 μmol/L Aβ_25–35_+10^−10^ mol/L triptolide group and 10^−10^ mol/L triptolide group. Scale bar, 10 μm. Fig b was the corresponding linear diagram of relative fluorescent intensity. Data were presented as the means ±mean ± S.E.M. n = 7/group. *P < 0.05, **P < 0.01 vs. control group. ^##^ *P* < 0.01 vs. 10 μmol/L Aβ_25–35_ group.

Staining with anti-LC3 is a recognized marker for autophagosomes. The fluorescence intensity and the number of bright fluorescent particles are related to the extent of lysosome acidity and these are used to predict the autophagy level. Additionally, in our study we addressed the distribution of LC3 in differentiated PC12 cells using confocal microscopy. As shown in Fig 7a, staining intensity and larger numbers of bright fluorescent particles in cells were visibly enhanced by Aβ_25–35_ treatment, indicating the presence of autophagosomes. The quantitative analysis in Fig 7b showed triptolide could inhibit the increase in the autophagy process induced by Aβ_25–35_(*P*<0.01). In addition, there was no significant difference with control cells in the triptolide only group (the raw data was showed in S2 Table).

### The level of LC3 in differentiated PC12 cells

**Fig 8:**
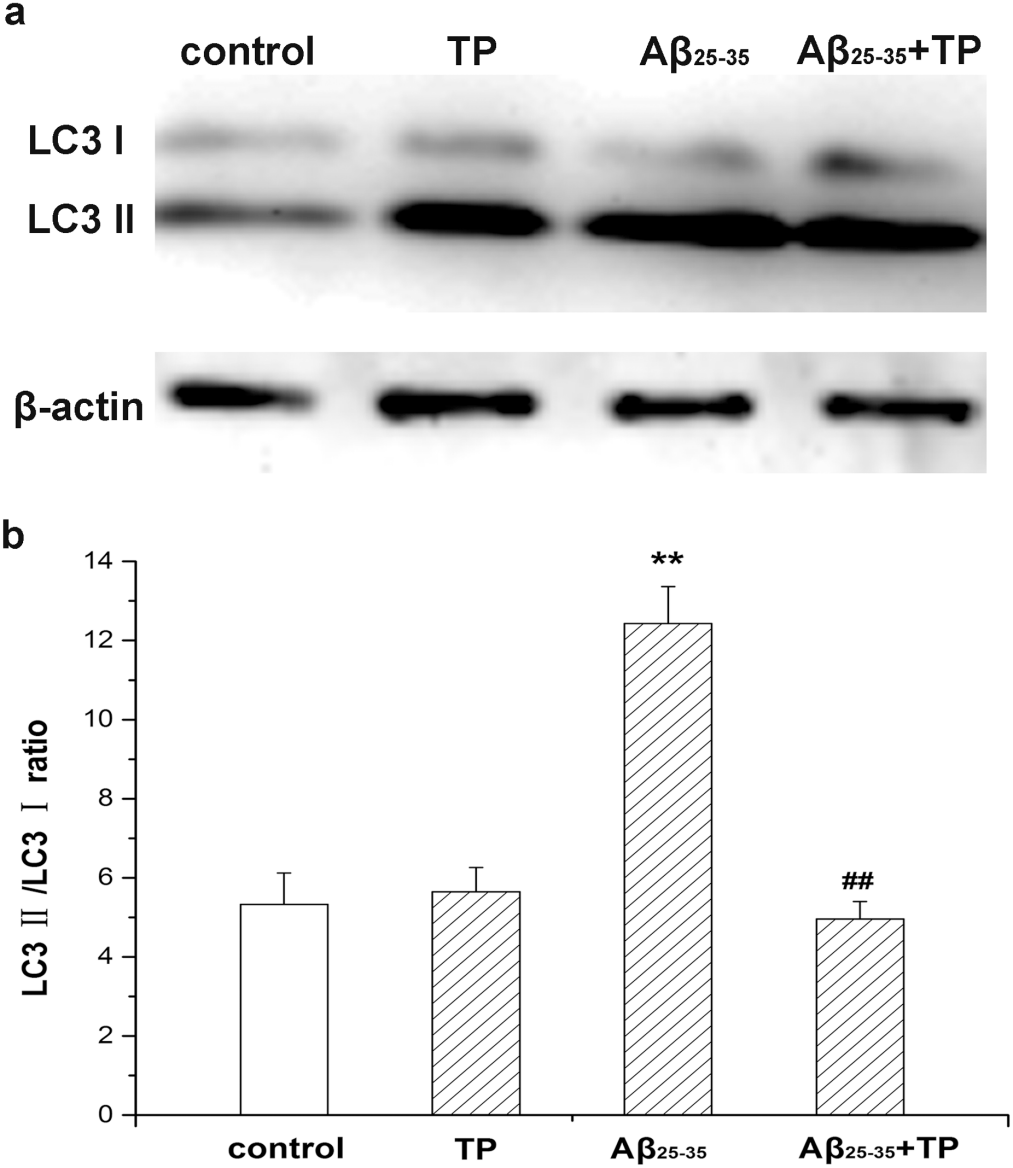
Ratio of LC3 II/LC3 I in differentiated PC12 cells. Differentiated PC12 cells were determined by measuring expression of LC3 protein, which was the autophagosome marker, using Western blot analysis. Cells were incubated with culture medium, 10^−10^ mol/L triptolide, 10 μmol/L Aβ_25–35_, and 10 μmol/L Aβ_25–35_+10^−10^ mol/L triptolide for 24 hours. The corresponding linear diagram of immunoblotting quantitation is shown in (b). The ratio of LC3 II/LC3 I was evaluated by densitometry analysis and data were expressed as folds of the control. Data represents the mean ± S.E.M. n = 3/group. *P < 0.05, **P < 0.01 vs. control group. ^#^ *P* < 0.05, ^##^ *P* < 0.01 vs. 10 μmol/L Aβ_25–35_ group.

To identify the level of autophagy, the expression of LC3 was examined by Western blot analysis. Conjugated LC3, called LC3 II, is the canonical marker of autophagosomes. During autophagy, LC3 I is cleaved to LC3 II, while the LC3 II/LC3 I ratio increases. As a result, we found that the LC3 II/LC3 I ratio of Aβ_25–35_-treated cells increased compared to the control groups (the raw figure was showed in S1 Fig). When incubated with triptolide, the LC3 II/LC3 I ratio in differentiated PC12 cells was significantly decreased (Fig 8). Our experiments suggest that autophagy was greatly and continually activated when differentiated PC12 cells were treated with Aβ_25–35_ and triptolide could inhibit this autophagy process.

### Effect of 3-MA and rapamycin

**Fig 9:**
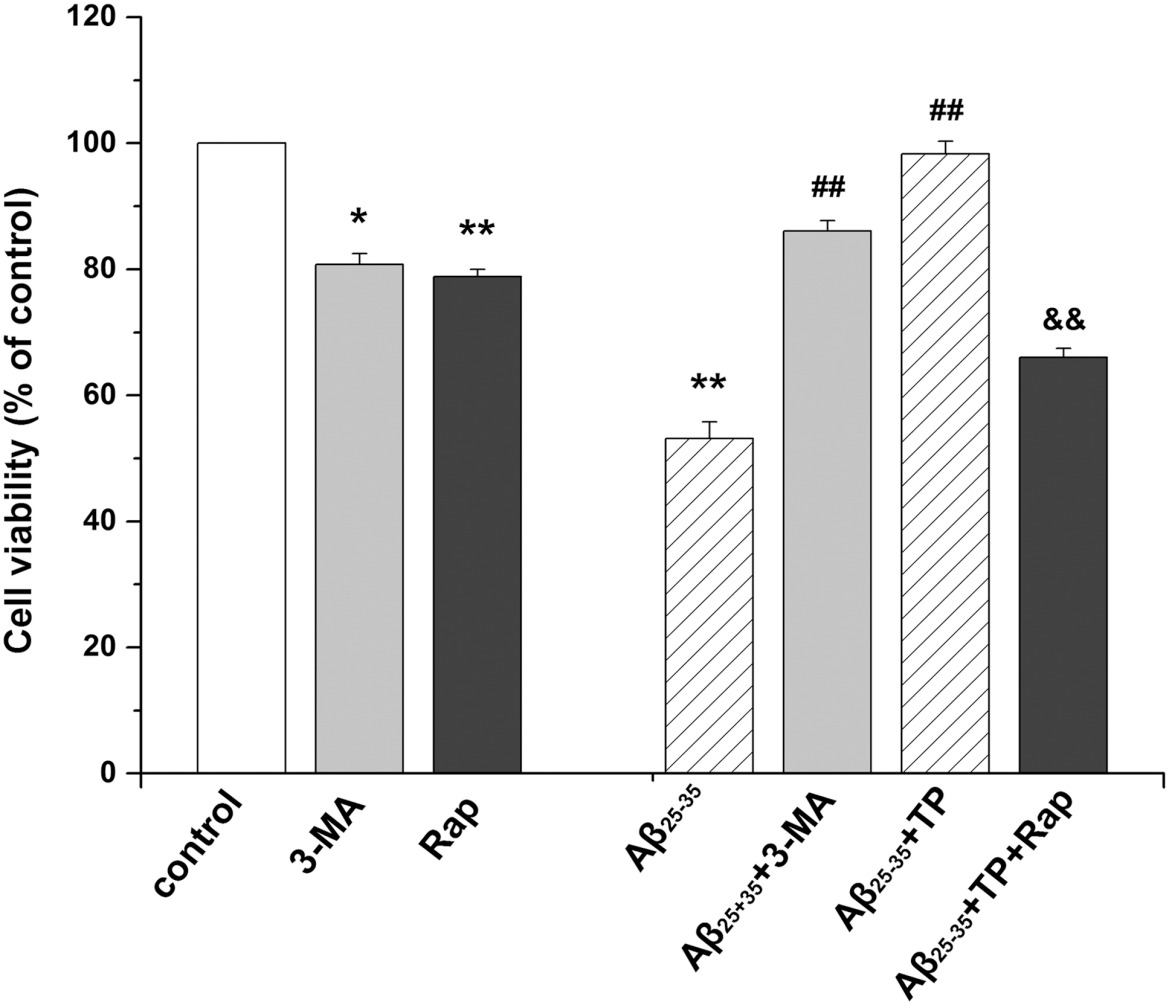
Effect of 3-MA and rapamycin. Differentiated PC12 cells were incubated with culture medium, 10 μmol/L Aβ_25–35_, 10 μmol/L 3-MA, 10ng/mL rapamycin, 10 μmol/L Aβ_25–35_+10 μmol/L 3-MA, 10 μmol/L Aβ_25–35_+10^−10^ mol/L triptolide, and 10 μmol/L Aβ_25–35_+10^−10^ mol/L triptolide +10ng/mL rapamycin, for 24 hours. The cell viability was determined by MTT. Data represents the mean ± S.E.M. n = 6/group. *P < 0.05, **P < 0.01 vs. control group. ^#^ *P* < 0.05, ^##^ *P* < 0.01 vs. 10 μmol/L Aβ_25–35_ group. ^&^ *P* < 0.05, ^&&^ *P* < 0.01 vs.10 μmol/L Aβ_25–35_+10^−10^ mol/L triptolide group.

As shown in Fig 9, the viability of PC12 cells was decreased in the rapamycin group (*P*<0.01) and 3-MA group *(P*<0.05). Cell viability was increased in the Aβ_25–35_+ 3-MA group, and there was a significant difference between the Aβ_25–35_ + 3-MA -treated group and Aβ-treated group (*P*<0.01). Then, we determined the effects of the autophagy activator rapamycin in Aβ_25–35_+ triptolide-treated differentiated PC12 cells. The viability of PC12 cells treated with rapamycin was reduced, and there was a significant difference between the Aβ_25–35_+triptolide+rapamycin-treated group and the Aβ_25–35_+triptolide-treated group (*P*<0.01).

## Discussion

AD is a devastating neurological disorder, and its hallmark pathologic characteristics are β-amyloid plaques, neurofibrillary tangles, and neurodegeneration. AD is a huge burden on society and patients’ families. Currently, due to rapidly aging populations, the prevalence of AD is growing exponentially. However, the pathogenesis of AD has not yet clearly been explained and there remains a dearth of effective treatments and cures. The deposition of Aβ was considered as a pathological hallmark of AD, and how to inhibit the cell apoptosis induced by Aβ was attracting considerable and increasing concern of the public [3].

Recent reports indicate that triptolide has neurotrophic and neuroprotective effects [40]. In our experiment, we investigated whether triptolide treatment could decelerate PC12 cells apoptosis, which treated with Aβ_25–35_. As a result, we found that Aβ_25–35_ was cytotoxic to differentiated PC12 cells and showed a concentration-dependent effect (Fig 1). In addition, the detection of apoptotic cells showed that the Aβ_25–35_ also induced the apoptosis of differentiated PC12 cells, which was significant between the control group and Aβ_25–35_ group (Fig 4). All of these results were consistent with previous studies indicating that Aβ_25–35_ could induce cell injury and death in differentiated PC12 cells [41, 42]. When treated with triptolide, the differentiated PC12 cells’ viability when incubated with Aβ_25–35_ increased. Additionally, triptolide also attenuated the apoptosis in differentiated PC12 cells induced by Aβ_25–35_, which implies that triptolide could protect differentiated PC12 cells with Aβ_25–35_. In addition, we also detected the effect of different concentrations of triptolide on differentiated PC12 cells because the safety of the drug is a crucial factor in clinical applications. As shown in Fig 3, differentiated PC12 cells were treated with different concentrations (10^−11^, 10^−10^, 10^−9^ mol/L) of triptolide for 24 hours, and there was no statistically significant difference in the cell viability between the control and triptolide group, which shows that our concentrations of triptolide were safe for differentiated PC12 cells.

Autophagy is an evolutionary conserved catabolic process used by eukaryotic cells for the degradation of damaged or superfluous proteins and organelles [43]. However, many scholars have found that excessive autophagy or autophagy activity perturbation were the arbiters in the formation and degradation of Aβ and tau protein [17] which might induce AD. Autophagy is regarded as a potential therapeutic target for AD.

Conventional wisdom suggests that ROS was one of the indicators of oxidative stress and an important factor in the apoptotic process [36, 44]. Accumulating evidence suggests that ROS act as signaling molecules involving a variety of intracellular processes. Furthermore, the intracellular excessive accumulation of ROS might mediate autophagy [45, 46]. For example, the addition of H_2_O_2_ could induce autophagy [47]. In some diseases, endogenous ROS levels were raised which then activated mitochondrial autophagy [47, 48]. In our research, the data of flow cytometry showed that the ROS level significantly increased when the differentiated PC12 cells were incubated with Aβ_25–35,_ and treatment with triptolide could decrease the intracellular ROS level in cells (Fig 5). We speculated that Aβ_25–35_ might induce autophagy through elevating the ROS level in differentiated PC12 cells and triptolide protected the cell by reducing the generation of ROS to weaken autophagy induced by Aβ_25–35_. However, ROS could induce autophagy or play an important role in the process, and various circumstances could cause an increase of intracellular ROS. Meanwhile, we also found that ROS generation of triptolide group was intermediate of the Aβ_25–35_ group and higher than control group, which meant that the pathway activated by ROS was just one of mediators in autophagy.

Acridine orange is a fluorescent dye which stains acidic compartments (such as lysosomes and autolysosomes) orange/red, while it stains cytoplasm and nuclei bright green. Acidic vacuoles, which are formed by autophagosomes fusing with lysosomes, can usually bind acridine orange in the process of cells’ autophagy. Therefore, acridine orange staining is often used to detect the occurrence of autophagy [49, 50]. Furthermore, in Fig 6, Aβ_25–35_-treated cells showed an obvious increase in orange fluorescence compared with that of the control group (*P*<0.01), which was indicated by the level of red fluorescence. And triptolide could decrease the level of acidic vesicular organelle during the autophagy process (*P*<0.01).

To further determine whether the autophagy was activated, we detected the expression of LC3, which is an autophagosome marker [51, 52]. LC3 exists in cytosolic LC3 I and phosphatidylethanolamine (PE)-conjugated LC3 II forms [53, 54]. Autophagosomes transport to the lysosomes for forming autolysosomes and LC3 II is degraded in autolysosomal lumen at the same time. Thus, as a marker of autophagosomal membranes, changes in the cellular LC3 II level reflect starvation-induced autophagic activity [52, 55]. To monitor autophagy during the treatment with triptolide, we measured the expression of LC3 by immunofluorescence and the LC3 II/LC3 I ratio by Western blot. Fig 7 shows a more intensive fluorescence was detected and the number of stained lysosomes was much higher than that in control cells (*P*<0.01). Additionally, there was a decrease in the autophagy process in triptolide-treated Aβ_25–35_ incubated cells (*P*<0.01). As shown in Fig 8, the LC3 II/LC3 I ratio increased with time during the incubation of Aβ_25–35_. A remarkable decrease of the LC3 II/LC3 I ratio was seen in triptolide-treated groups during incubation of Aβ_25–35_. The level of LC3 II is directly associated with the number of autophagosomes, therefore, the results show that triptolide inhibits the cytotoxicity of differentiated PC12 cells induced by Aβ_25–35_ via the autophagy pathway.

3-MA is an inhibitor of the class I phosphatidylinositol 3-kinase (PI3K) [56]. Further *in vitro* enzymatic assays show that 3-methyladenine also inhibits class III PI3K activity [57]. Recent studies indicate that PI3K, especially class III PI3K, is essential in the regulation of autophagy. 3-MA inhibits autophagy by inhibition of these enzymes. Rapamycin is commonly used as a specific activator of autophagic sequestration. The literature observes that the increase is accompanied by a significant increase in autophagy due to the mammalian target of the rapamycin (mTOR) pathway inhibition [58]. As a result, cell viability was increased by 3-MA, and there was a significant difference between the Aβ_25–35_ +3-MA-treated group and Aβ-treated group (*P*<0.01). 10 ng/mL of rapamycin could markedly reduce the viability compared with the triptolide-treated group (*P*<0.01) (Fig 9). These results further confirm that autophagy plays an important role in the protection of triptolide on differentiated PC12 cells.

In summary, we discovered that Aβ_25–35_ could induce cell death and cell apoptosis via the activation of autophagy in differentiated PC12 cells. Furthermore, triptolide has a protective effect against Aβ_25–35_-induced cytotoxicity in differentiated PC12 cells by inhibiting the autophagy pathway. In our further experiments, we will detect the molecular signaling mechanism of oxidative stress (because of the generation of ROS in triptolide group) and investigate whether triptolide inhibits autophagy in an mTOR-dependent manner (because of the function of rapamycin), and we will also check the effect of triptolide in primary cells and in *in vivo* models to compare the difference of the molecular signaling mechanism of autophagy in pathogenesis of AD between *in vivo* and *in vitro*. These results may suggest that triptolide will be a promising tool for AD research. It will be helpful to provide an interesting view of the potential application of triptolide or TWHF in the future study of AD.

## Supporting Information

S1 Fig Raw figure of Western blot analysis. Fig a was the expression of LC3 I and LC3 II. Fig b was the expression of β-actin on the same Western blot sample.
(TIF)

S1 Table Raw data of acridine orange staining. For the quantitation analysis, red fluorescent intensity and green fluorescent intensity were quantified using IPP 6.0.
(DOCX)

S2 Table Raw data of the expression of LC3 with immunofluorescence staining. For the quantitation analysis, fluorescent intensity was quantified using IPP 6.0.
(DOCX)
