# Supplementary figures and images for: Triptolide Inhibited Cytotoxicity of Differentiated PC12 Cells Induced by Amyloid-Beta25–35 via the Autophagy Pathway
Source: PLoS One. 2015 Nov 10;10(11):e0142719. doi: 10.1371/journal.pone.0142719 (PMC4640509; doi:10.1371/journal.pone.0142719)

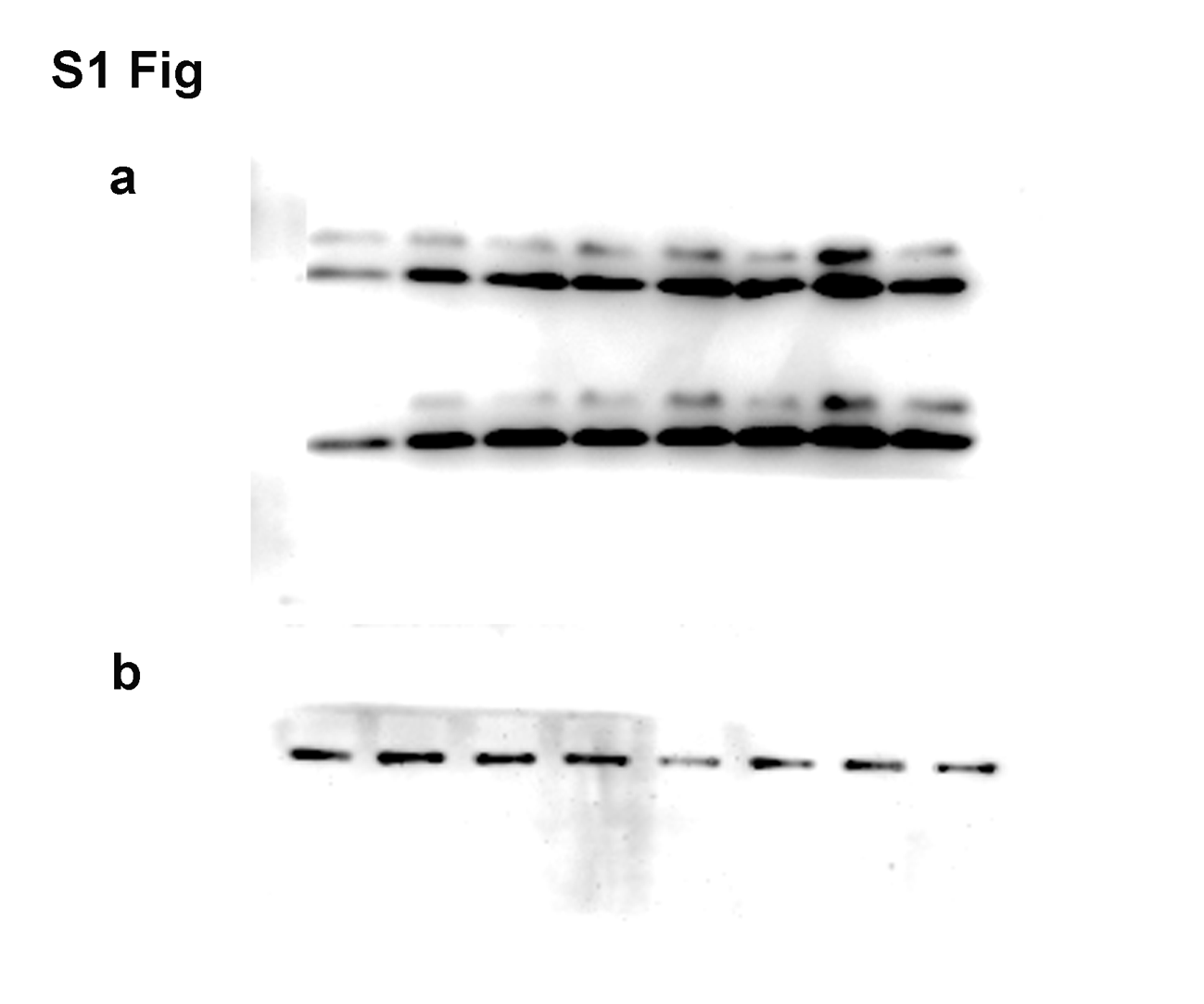

Supplement: S1 Fig — Fig a was the expression of LC3 I and LC3 II. Fig b was the expression of β-actin on the same Western blot sample. (TIF) [file pone.0142719.s001.tif]
