# Supplementary material for: Triptolide Inhibited Cytotoxicity of Differentiated PC12 Cells Induced by Amyloid-Beta25–35 via the Autophagy Pathway
Source: PLoS One. 2015 Nov 10;10(11):e0142719. doi: 10.1371/journal.pone.0142719 (PMC4640509; doi:10.1371/journal.pone.0142719)
Supplement: S1 Table — For the quantitation analysis, red fluorescent intensity and green fluorescent intensity were quantified using IPP 6.0. (DOCX) [file pone.0142719.s002.docx]

**S1 Table. Raw data of acridine orange staining. (n=7)**

|  | **Red fluorescence** | | | **Green fluorescence** | | |
| --- | --- | --- | --- | --- | --- | --- |
|  | **control** | **10μmol/L Aβ_25-35_** | **10μmol/L Aβ_25-35_ +**  **10^-10^mol/L triptolide** | **control** | **10μmol/L Aβ_25-35_** | **10μmol/L Aβ_25-35_ +**  **10^-10^mol/L triptolide** |
| **raw** | 0.02089445 | 0.05466732 | 0.01751144 | 0.0919724 | 0.06374294 | 0.06500992 |
|  | 0.02217008 | 0.07291597 | 0.03168089 | 0.09412483 | 0.02976462 | 0.15616494 |
| **data** | 0.03184229 | 0.06469345 | 0.03483112 | 0.1696193 | 0.03500379 | 0.07326626 |
|  | 0.04027978 | 0.05400795 | 0.02211822 | 0.12797298 | 0.05129721 | 0.08115142 |
|  | 0.02504353 | 0.063887 | 0.0306997 | 0.11801504 | 0.04757573 | 0.05506737 |
|  | 0.0276466 | 0.07105599 | 0.02587956 | 0.09305305 | 0.07910895 | 0.13012522 |
|  | 0.03410026 | 0.07400549 | 0.02338957 | 0.10499479 | 0.08280621 | 0.13163686 |
| **mean±S.E.M** | **0.0289±**  **0.00263** | **0.0650±**  **0.00312** | **0.0266±**  **0.00231** | **0.1143±**  **0.01058** | **0.0556±**  **0.00777** | **0.0989**±  **0.01493** |
