# Supplementary material for: Triptolide Inhibited Cytotoxicity of Differentiated PC12 Cells Induced by Amyloid-Beta25–35 via the Autophagy Pathway
Source: PLoS One. 2015 Nov 10;10(11):e0142719. doi: 10.1371/journal.pone.0142719 (PMC4640509; doi:10.1371/journal.pone.0142719)
Supplement: S2 Table — For the quantitation analysis, fluorescent intensity was quantified using IPP 6.0. (DOCX) [file pone.0142719.s003.docx]

**S2 Table. Raw data of the expression of LC3 with immunofluorescence staining. (n=7)**

|  | **control** | **10μmol/L Aβ_25-35_** | **10μmol/L Aβ_25-35_ +**  **10^-10^mol/L triptolide** | **10^-10^mol/L triptolide** |
| --- | --- | --- | --- | --- |
|  | 0.03083756 | 0.06152339 | 0.02042588 | 0.02296453 |
| **raw** | 0.02509284 | 0.07563675 | 0.03162755 | 0.0248278 |
|  | 0.02374087 | 0.06781213 | 0.01999234 | 0.03354805 |
| **data** | 0.02489797 | 0.07327362 | 0.02442774 | 0.03766899 |
|  | 0.0240714 | 0.05462495 | 0.02526768 | 0.02180366 |
|  | 0.02344435 | 0.07283059 | 0.03583901 | 0.02541549 |
|  | 0.03312863 | 0.05830833 | 0.02212043 | 0.02155569 |
| **mean±S.E.M** | **0.0265±0.00146** | **0.0663±0.00310** | **0.0257±0.00225** | **0.0268±0.00237** |
